# Coadministration of DPP-4 inhibitor and insulin therapy does not further reduce the risk of cardiovascular events compared with DPP-4 inhibitor therapy in diabetic foot patients: a nationwide population-based study

**DOI:** 10.1186/s13098-018-0378-6

**Published:** 2018-10-17

**Authors:** Yi-Hsuan Lin, Yu-Yao Huang, Yi-Ling Wu, Cheng-Wei Lin, Pei-Chun Chen, Chee Jen Chang, Sheng-Hwu Hsieh, Jui-Hung Sun, Szu-Tah Chen, Chia-Hung Lin

**Affiliations:** 1 Division of Endocrinology and Metabolism, Department of Internal Medicine, Chang Gung Memorial Hospital, 5, Fusing St, Gueishan Township, Taoyuan County, 333 Taiwan; 2 Department of Medical Nutrition Therapy, Chang Gung Memorial Hospital, Linkou, Taiwan; 3 Research Services Center for Health Information, Chang Gung University, Taoyuan, Taiwan; 4 Department of Public Health, China Medical University, Taichung, Taiwan; 5 Graduate Institute of Clinical Medical Sciences, Chang Gung University, Taoyuan, Taiwan; 6 Clinical Informatics and Medical Statistics Research Center, Chang Gung University, Taoyuan, Taiwan; 7 Department of Cardiology, Chang Gung Memorial Hospital, Linkou, Taiwan; 8 Department of Chinese Medicine, Chang Gung University, Taoyuan, Taiwan

**Keywords:** Diabetic foot, Dipeptidyl peptidase-4 inhibitor-based therapy, Insulin therapy, Cardiovascular complications

## Abstract

**Background:**

The effect of combined insulin and dipeptidyl peptidase-4 inhibitor (DPP4i) therapy on major adverse cardiovascular events (MACEs) in patients with diabetic foot is unclear.

**Methods:**

We conducted this nationwide cohort study using longitudinal claims data obtained from the Taiwan National Health Insurance program and included 19,791 patients with diabetic foot from 2007 to 2014. Patients receiving DPP4i-based therapy and/or insulin-based therapy after a diagnosis of diabetic foot were categorized into combined, DPP4i- or insulin-based groups, respectively. The risk of MACEs including nonfatal myocardial infarction, nonfatal stroke, cardiac death, and heart failure was assessed using Cox proportional hazards analysis and propensity score matching.

**Results:**

Among the 19,791 patients with diabetic foot (mean age, 58.8 years [SD, 12.5]; men, 51.2%), 6466 received DPP4i-based therapy, 1925 received insulin-based therapy, and 11,400 received combined DPP4i and insulin therapy. The DPP4i-based and insulin-based groups had a lower risk of MACEs (HR 0.53, 95% CI 0.50–0.57 DPP4i only; HR 0.89, 95% CI 0.81–0.97 insulin only) than the combined group. After propensity score matching, the incidence of all complications in the DPP4i-based group was still significantly lower than that in the combined group (HR 0.55, 95% CI 0.51–0.59 for MACEs; HR 0.32, 95% CI 0.24–0.42 for nonfatal myocardial infarction; HR 0.70, 95% CI 0.63–0.78 for nonfatal stroke; HR 0.22, 95% CI 0.13–0.38 for cardiac death; HR 0.22, 95% CI 0.19–0.25 for any death; HR 0.16, 95% CI 0.13–0.20 for amputation). In the diabetic foot patients with end-stage renal disease (ESRD), the benefit of a lower incidence of MACEs in the DPP4i-based group disappeared (HR 0.77, 95% CI 0.58–1.08).

**Conclusions:**

This study demonstrated that the patients with diabetic foot receiving DPP4i-based therapy had a lower risk of MACEs than those receiving combined therapy with DPP4i and insulin, but that the effect disappeared in those with concurrent ESRD.

**Electronic supplementary material:**

The online version of this article (10.1186/s13098-018-0378-6) contains supplementary material, which is available to authorized users.

## Background

As the worldwide prevalence of diabetes mellitus increases, the incidence rates of concomitant complications such as renal failure, coronary artery disease, stroke, and lower limb amputation are also increasing [1]. The lifetime incidence of diabetic foot among patients with diabetes mellitus is close to 25% [2], and nearly half of these patients have peripheral artery disease [3]. Statistical data in Taiwan from 2000 to 2009 show an amputation rate of approximately 30% in patients admitted to hospital for diabetic foot treatment [4]. A previous study reported that the intensive control of glycaemia with insulin or sulfonylurea can decrease microvascular complications, and further follow-up demonstrated a benefit regarding macrovascular events over the course of more than 10 years [5, 6]. However, some researchers have found that insulin therapy may have a proatherogenic effect and increase oxidative stress, thereby worsening cardiovascular outcomes [7–10]. On the other hand, other studies have shown anti-inflammatory and antiangiogenic effects with insulin usage for diabetic wounds [11]. An increasing number of antihyperglycemic medications with different mechanisms have become available, including incretin-based therapies [12]. Fadini et al. [13] demonstrated that vaso-protective endothelial progenitor cells proliferated after 4 weeks of sitagliptin treatment. In addition, Kawanami et al. reported that incretin-based medications, including a dipeptidyl peptidase-4 (DPP-4) inhibitor and a glucagon-like peptide-1 (GLP-1) receptor agonist, showed anti-inflammatory and antioxidant effects and also ameliorated atherosclerosis [14]. These results suggest that diabetic vascular complications are due to hyperglycemia and the pleiotropic action of antidiabetic drugs associated with atherosclerosis and endothelial dysfunction [15].

Several studies have also reported the benefits of glycaemic control with DPP4i-based therapy in combination with insulin. Chen et al. [16] conducted a meta-analysis of seven randomized controlled trials of combination therapy with a DPP-4 inhibitor and insulin compared with insulin with or without other oral antidiabetic drugs, and found a significantly greater reduction in HbA1c and 2-h postprandial glucose levels in patients treated with the DPP-4 inhibitor combined with insulin. Several other studies have reported that the combination of insulin and DDP4i therapy led to a greater decrease in HbA1c level than insulin or DPP4i alone [17–19]. In addition, DPP4i-based therapy has been shown to reduce the incidence of diabetic vascular complications [20–22]. Because these complications can result in diabetic foot, decreased quality of life, and be a strain on medical resources, clarification of the medical effects of antidiabetic agents on diabetic complications is important.

One of the most common complications related to diabetes is chronic kidney disease [23], and the condition limits the choice of antidiabetic drug. Furthermore, in patients with diabetic foot ulcers, renal function has been reported to be worse than in patients without foot ulcers [24]. For these reasons, insulin- and incretin-based therapies are commonly used for glycemic control in patients with diabetic foot [25, 26]. However, according to a previous study, cardiovascular outcomes could not be explained simply by glycemic control [27]. In addition, the effect of insulin- and DPP4i-based therapy on diabetic vascular complications in patients with diabetic foot is still largely unclear. Studies on insulin therapy combined with a DPP4i have emphasized glycaemic control [28] and less on adverse cardiovascular outcomes, especially in patients with diabetic foot. Therefore, we conducted this nationwide population-based cohort study using the National Health Insurance Research Database (NHIRD) in Taiwan to analyze the relationship between the incidence and prognosis of major adverse cardiovascular events (MACEs) in patients receiving a combination of DPP4i- and insulin-based therapy. The aim of this study was to investigate whether using combined insulin- and DPP4i-based therapy in patients with type 2 diabetes and diabetic foot would have a beneficial effect on MACEs.

## Methods

### Source of data

We conducted this retrospective cohort study using longitudinal claims data from the Taiwan National Health Insurance program from 2007 to 2014. Taiwan implemented its single-payer compulsory National Health Insurance program in 1995, and it currently covers 99% of the Taiwanese population and reimburses for outpatient visits, hospital admissions, and prescriptions. All contracted institutions must file claims according to standard formats, which are later archived in the NHIRD. This study was approved by the Institutional Review Board of Chang Gung Memorial Hospital, Taiwan.

### Study population

**Fig. 1:**
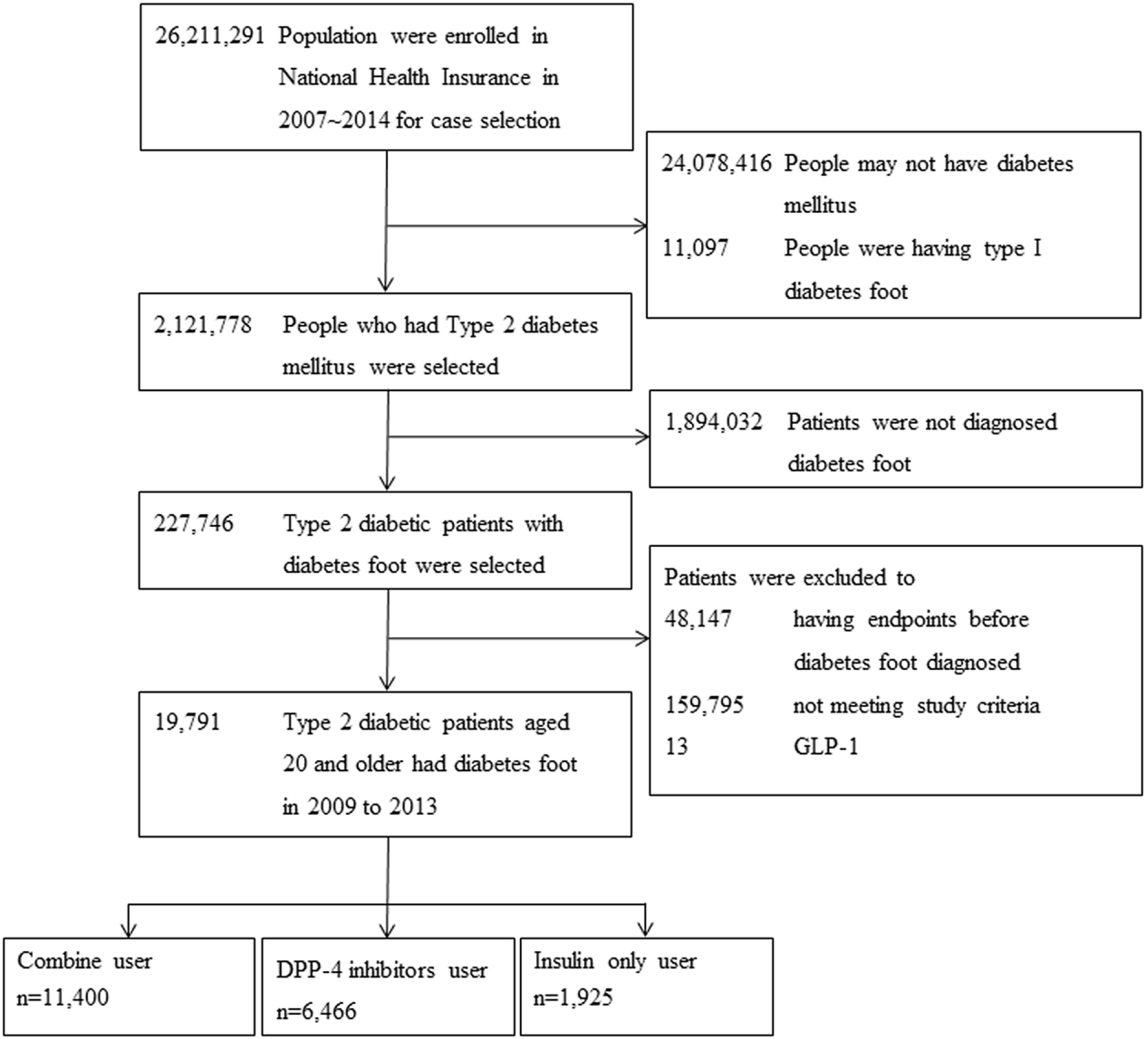
Flowchart of study population selection

We identified all patients with diabetes (aged ≥ 20 years) who had any form of diabetic foot disease (International Classification of Diseases, Ninth Revision (ICD-9), codes 250.70, 440.2, 443.9, peripheral artery disease; 040.0, gas gangrene; 785.4, gangrene; 730.07, acute osteomyelitis, ankle and foot; 730.17, chronic osteomyelitis, ankle and foot; 730.27, unspecified osteomyelitis, ankle and foot; 730.97, unspecified infection of bone, ankle and foot; 707.14, ulcer of the heel and midfoot; 707.15, ulcer of other part of the foot; 707.1, ulcer of lower limbs, except pressure ulcer; 680.7, carbuncle and furuncle of the foot; 682.7, cellulitis and abscess of the foot, except toes; 681.1, cellulitis and abscess of toes; and 681.10, cellulitis and abscess of toes, unspecified as well as V49.71–V49.77 (great toe amputation status, other toe(s) amputation status, foot amputation status, ankle amputation status, below the knee amputation status, above the knee amputation status, hip amputation status) and V52.1, fitting and adjustment of artificial leg (complete or partial) or ICD-9 procedure codes 84.11–84.17 (amputation of a toe, amputation through the foot, disarticulation of the ankle, amputation of the ankle through malleoli of the tibia and fibula, other amputation below the knee, disarticulation of the knee, amputation above the knee) after a diagnosis of diabetes (Additional file 1: Table S1). Patients with diabetes mellitus were identified in the NHIRD from 2009 to 2013 on the basis of the following criteria: (1) hospital admission for diabetes-related illness (ICD-9 code 250.xx), and (2) three or more outpatient codes within a calendar year for diabetes-related illness. Patients who were documented as having type 1 diabetes (ICD-9 code 250.x1 or 250.x3) in the catastrophic illness registry were excluded. We also excluded patients who had a MACE prior to diabetic foot disease (Fig. 1). This nationwide study included all available and eligible patients.

Patients receiving combined DPP4i and insulin therapy for at least 7 days after receiving a diagnosis of diabetic foot disease were categorized in the combined therapy group, and those receiving at least 7 days of either DPP4i therapy or insulin therapy were assigned to the DPP4i-based group or insulin-based group, respectively. Patients receiving any therapy but for less than 7 days were excluded. The first date of therapy during the study period was defined as the index date.

DPP4i-based therapy and insulin-based therapy were defined as receiving available medications during the study period (2007–2014) in Taiwan. The DPP4i-based therapy included DPP-4 inhibitors (sitagliptin, saxagliptin, linagliptin, vildagliptin), and the insulin-based therapy included basal insulin (detemir, glargine) and non-basal insulin [short acting insulin, ultra-short acting insulin, pre-mixed insulin, Neutral protamine Hagedorn (NPH) insulin]. The use of common antithrombotic medications for patients with diabetic foot including aspirin, cilostazol, warfarin, and clopidogrel was also recorded for analysis.

Comorbidities were identified using ICD-9 codes based on coding for any admission and on coding for more than two outpatient visits before the diagnosis of diabetic foot disease. Patients treated with DPP4i-based therapy and insulin-based therapy after a diagnosis of diabetic foot disease were matched to patients treated with DPP4i-based therapy or insulin-based therapy by propensity score matching in a 1:1 ratio on the basis of age, sex, hypertension, hyperlipidemia, nephropathy, retinopathy, peripheral neuropathy, antithrombotic therapy, and the index date.

### Main outcome measures

The primary outcome of this study was the first MACE [including nonfatal myocardial infarction (MI), nonfatal stroke, cardiac death, and heart failure]. The secondary outcomes were nonfatal MI (ICD-9 code 410), nonfatal stroke (ICD-9 codes 433–437), cardiac death, death resulting from any cause, and amputation (ICD-9 codes 84.11–84.17) and heart failure for admission (ICD-9 codes 428.xx). Causes of death were defined according to the death registry database. The follow-up period for outcome events was calculated from the index date to the date of first MACE, death during any hospitalization, last medical claim, or the end of 2014, whichever came first.

### Statistical analysis

The baseline characteristics of the three treatment groups were compared using analysis of variance for continuous variables and the chi-squared test for categorical variables. Associations between diabetic therapy and MACEs in the diabetic patients with diabetic foot disease were analyzed using a Cox proportional hazards model, and results are presented as unadjusted hazard ratios (HRs) with 95% confidence intervals (95% CIs). To reduce differences in baseline characteristics among the groups, we conducted propensity score matching with two contrasts separately: DPP4i-based versus combined therapy, and insulin-based versus combined therapy. For each of the two comparisons, we used separate logistic regression models to estimate a propensity score, and then created matched pairs. The characteristics and Cox proportional hazards model for adjusted HRs and 95% CIs are also presented for the matched pairs. In the two logistic regression models, we adjusted for confounding factors including age, sex, hypertension, hyperlipidemia, nephropathy, retinopathy, peripheral neuropathy, antithrombotic therapy, and the index date. We also performed subgroup analyses stratified by end-stage renal disease (ESRD), antithrombotic medication, and insulin type. All statistical analyses were performed using SAS statistical software version 9.4 (SAS Institute Inc., Cary, NC). A two-sided *P* value of < 0.05 was considered to be statistically significant.

## Results

### Characteristics of the study population

From 1 January, 2007 to 31 December, 2014, a total of 26,211,291 individuals were enrolled in the Taiwan National Health Insurance program. We excluded from our analysis 24,078,416 people without diabetes mellitus and 11,097 people with type 1 diabetes. Among the remaining 2,121,778 patients with type 2 diabetes, 1,894,032 and 48,147 were further excluded because they did not have a diagnosis of diabetic foot and had endpoints before a diagnosis of diabetic foot, respectively. Another 159,795 people were excluded because they did not meet the study criteria and 13 people were excluded due to GLP-1 usage. Thus, our study included a total of 19,791 patients with type 2 diabetes aged 20 years and older with a diagnosis of diabetic foot. Of these patients, 6466 were classified into the DPP4i-based therapy group, 1925 into the insulin-based therapy group, and 11,400 into the combined DPP4i- and insulin-based therapy group (Fig. 1). The demographic characteristics including sex, age, comorbidities, and antithrombotic drug use are summarized in Additional file 2: Table S2. Most patients with diabetic foot were in the 50–59 year age group (31.0% in total and 30.0%, 33.1%, and 30.1% in the combined, DPP4i-based, and insulin-based groups, respectively), and most of the patients (57.6%) received combination therapy with a DPP4i and insulin. The proportions of retinopathy, nephropathy, neuropathy, peripheral artery disease (PAD) and use of antithrombotic drugs were higher in the combined therapy group than in the other two groups. The rates of hypertension and hyperlipidemia were higher in the DPP4i-based group than in the other two groups.

### Analysis of MACEs in the patients with diabetic foot

**Fig. 2:**
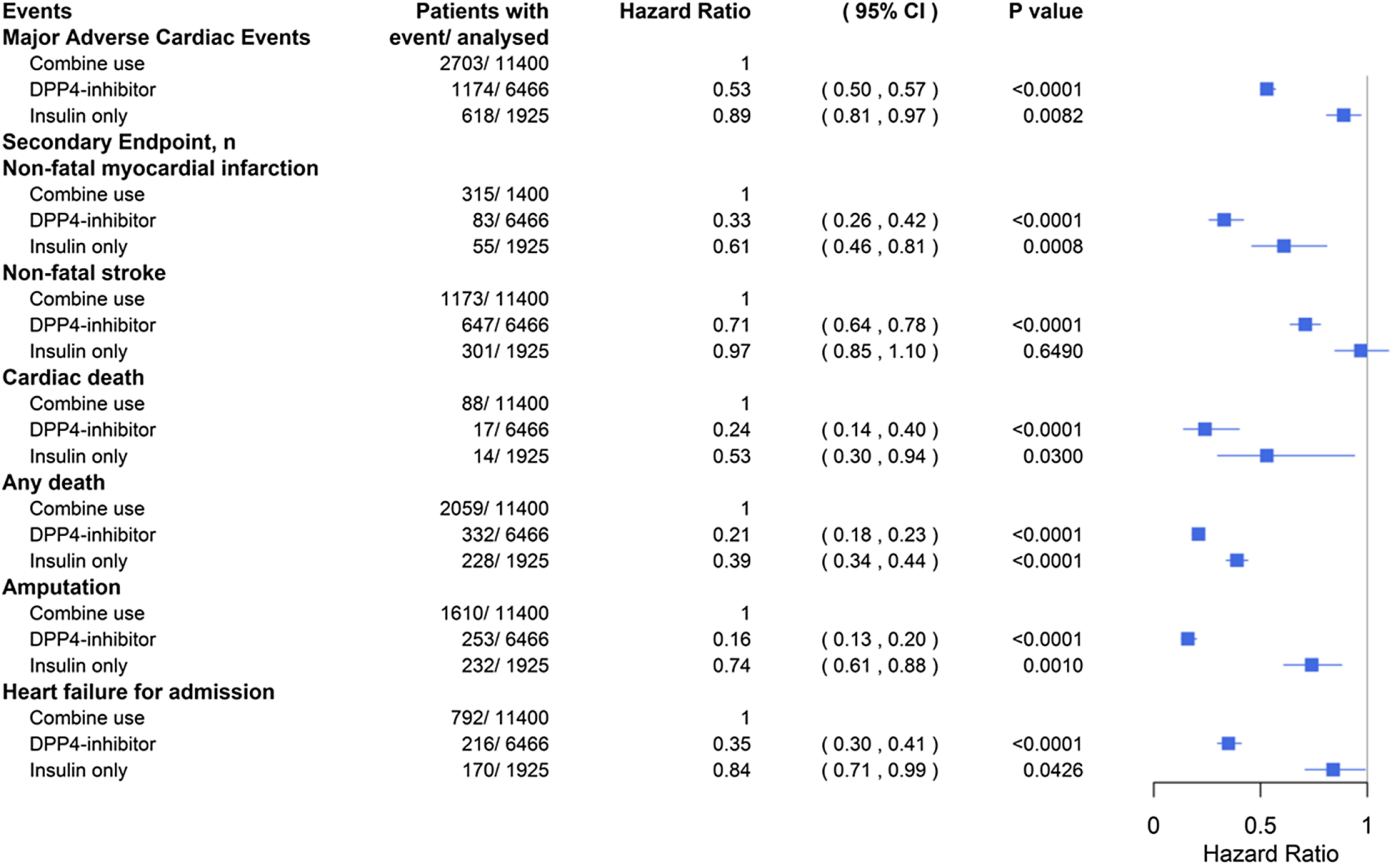
Cox proportional hazard models for primary and secondary endpoints in the patients with diabetes mellitus and diabetic foot. The probability of mortality and cardiovascular outcomes in the patients with diabetes mellitus and diabetic foot is shown for the DPP4i-only or insulin-only group versus the combined therapy group. The effects of DPP4i-only and insulin-only therapy on MACEs, nonfatal MI, nonfatal stroke, cardiac death, death resulting from any cause, amputation and heart failure for admission were analyzed individually. *DPP4i* dipeptidyl peptidase-4 inhibitor, *HR* hazard ratio, *CI* confidence interval, *MACE* major adverse cardiac event, *MI* myocardial infarction

Cox proportional hazards models were used to analyze the primary and secondary endpoints among the three groups, with the combined insulin- and DPP4i-based therapy group serving as the reference group (Fig. 2). There was a lower risk in the DPP4i-based and insulin-based groups than in the combined treatment group for MACEs (HR 0.53, 95% CI 0.50–0.57 for DPP4i-based; HR 0.89, 95% CI 0.81–0.97 for insulin-based), nonfatal MI (HR 0.33, 95% CI 0.26–0.42; HR 0.61, 95% CI 0.46–0.81), cardiac death (HR 0.24, 95% CI 0.14–0.40; HR 0.53, 95% CI 0.30–0.94), any death (HR 0.21, 95% CI 0.18–0.23; HR 0.39, 95% CI 0.34–0.44), amputation (HR 0.16, 95% CI 0.13–0.20; HR 0.74, 95% CI 0.61–0.88), and heart failure for admission (HR 0.35, 95% CI 0.30–0.41; HR 0.84, 95% CI 0.71–0.99). For nonfatal stroke, the DPP4i-based group but not the insulin-based group had a lower risk than the combined therapy group (HR 0.71, 95% CI 0.64–0.78; HR 0.97, 95% CI 0.85–1.10, respectively).

We used separate logistic regression models to estimate propensity scores, and created matched pairs with confounders of sex, age, antithrombotic drug use, hypertension, hyperlipidemia, nephropathy, retinopathy, peripheral neuropathy and PAD. The baseline characteristics of the patients with diabetic foot after propensity score matching are shown in Additional file 3: Table S3. The combined therapy group had the highest rate of ESRD, followed by the insulin-based group. The DPP4i-based group had the fewest patients with ESRD.

**Fig. 3:**
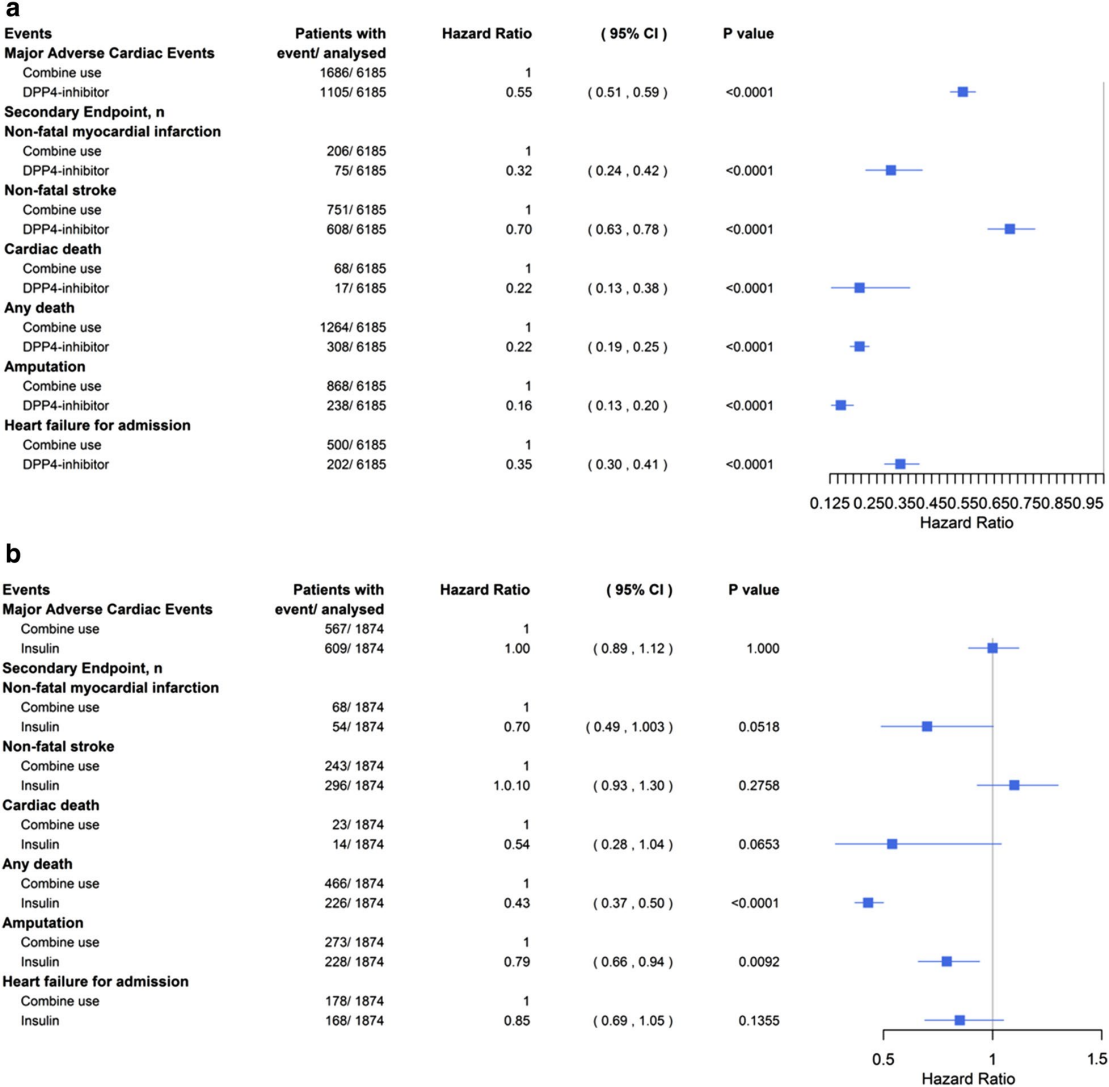
The probability of mortality and cardiovascular outcomes in the patients with diabetes mellitus and diabetic foot in the DPP4i-only group or insulin-only group versus the combined therapy group by propensity score matching. The effects of DPP4i-only (**a**) and insulin-only therapy (**b**) on major adverse cardiac events, nonfatal myocardial infarction, nonfatal stroke, cardiac death, death resulting from any cause, amputation and heart failure for admission were analyzed individually. *DPP4i* dipeptidyl peptidase-4 inhibitor, *CI* confidence interval

Cox proportional hazard models for primary and secondary endpoints were analyzed using propensity score matching (Fig. 3). The incidence of all complications in the DPP4i-based group was still significantly lower than that in the combined therapy group (HR 0.55, 95% CI 0.51–0.59 for MACEs; HR 0.32, 95% CI 0.24–0.42 for nonfatal MI; HR 0.70, 95% CI 0.63–0.78 for nonfatal stroke; HR 0.22, 95% CI 0.13–0.38 for cardiac death; HR 0.22, 95% CI 0.19–0.25 for any death; HR 0.16, 95% CI 0.13–0.20 for amputation; HR 0.35, 95% CI 0.30–0.41 for heart failure for admission). In the insulin-based group, only the risk of any death (HR 0.43, 95% CI 0.37–0.50) and amputation (HR 0.79, 95% CI 0.66–0.94) were lower than those in the combined therapy group.

**Fig. 4:**
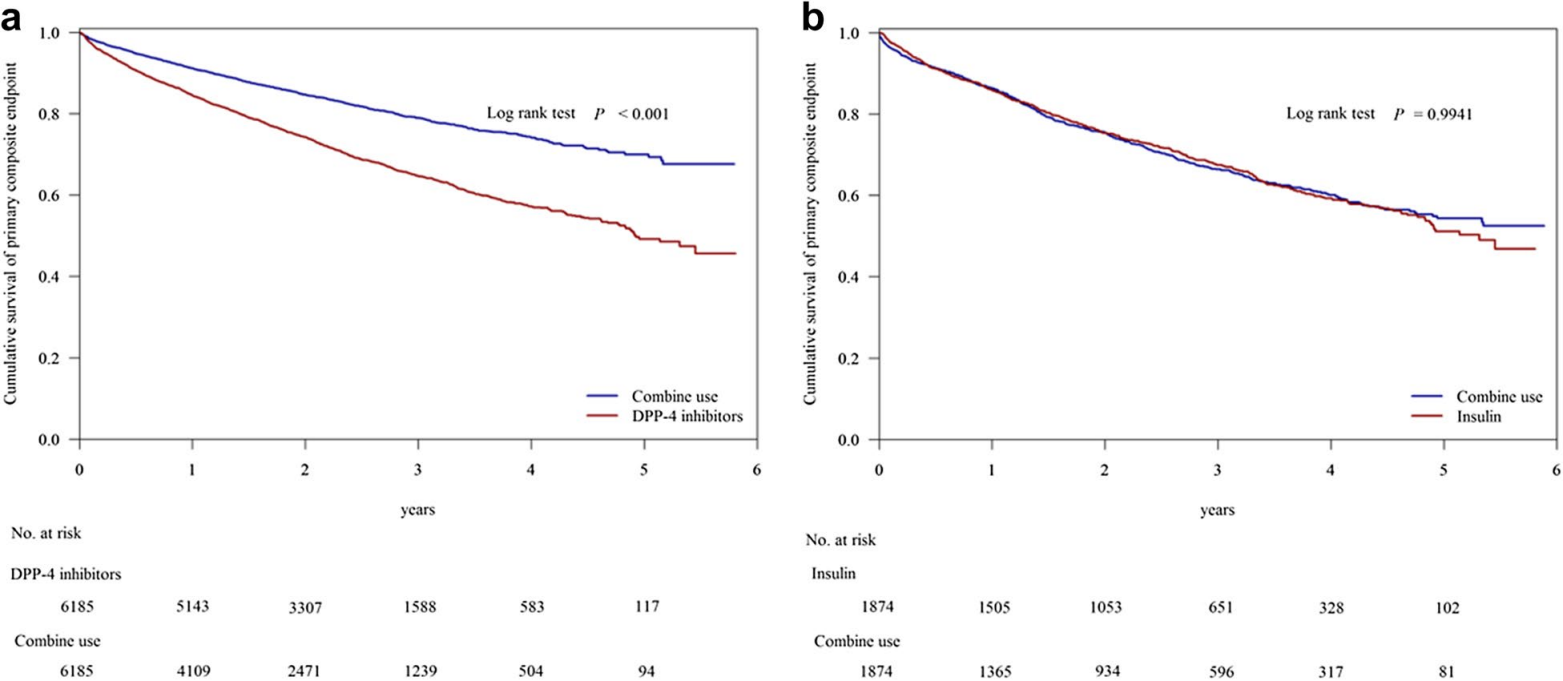
MACE-free survival rates in the patients with diabetes mellitus and diabetic foot. The primary outcome was estimated using Cox regression models stratified according to trial and history of MACEs for the DPP4i-only group (**a**) or the insulin-only group (**b**) versus the combined therapy group. *MACE* major adverse cardiac event, *DPP4i* dipeptidyl peptidase-4 inhibitor

In survival analysis, the risk of MACEs was significantly lower in the DPP4i-based group than in the combined therapy group (log-rank test P < 0.001) (Fig. 4). The difference between the insulin-based group and combined therapy group was not significant after up to 6 years of follow-up (log-rank test P = 0.994).

### Subgroup analysis

**Fig. 5:**
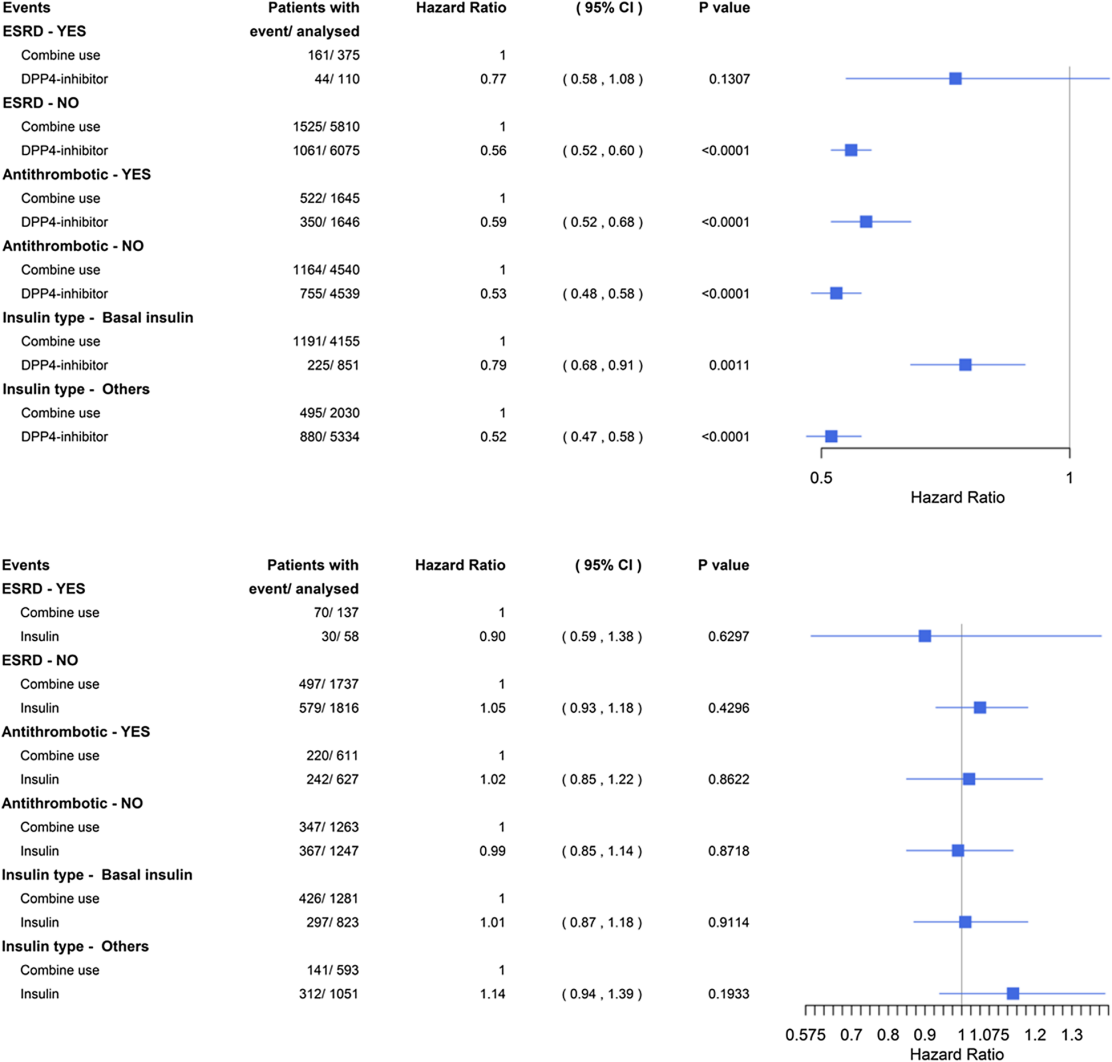
Cox proportional hazard regression analyses were performed for subgroups of patients with respect to the primary MACE outcome (including nonfatal myocardial infarction, nonfatal stroke, cardiac death, and heart failure). P values signify the differences between groups. *CI* confidence interval, *ESRD* end-sage renal disease, *MACE* major adverse cardiac event, *DPP4* dipeptidyl peptidase-4

We further divided the participants with diabetic foot into subgroups according to ESRD, antithrombotic drug use, and insulin type (Fig. 5). Comparing the DPP4i-based group with the combined therapy group, we found a lower incidence of MACEs regardless of antithrombotic drug use or different insulin type. However, in the diabetic foot patients with ESRD, the lower risk of MACEs in the DPP4i-based group disappeared (HR 0.77, 95% CI 0.58–1.08 vs. HR 0.56, 95% CI 0.52–0.60 in those with ESRD vs. those without ESRD, respectively). However, the insulin-based group did not have a significantly lower incidence of MACEs than the combined therapy group in subgroup analysis.

Although the incidence of MACEs in the DPP4i-based group was significantly lower than that in the combined therapy group regardless of insulin type, the difference between the DPP4i-based group and the DPP4i-based group combined with the non-basal insulin group was greater than that between the DPP4i-based group and the DPP4i with basal insulin-only group (HR 0.52, 95% CI 0.47–0.58 vs. HR 0.79, 95% CI 0.68–0.91 in those receiving combined therapy with non-basal vs. basal insulin-only therapy, respectively).

## Discussion

In diabetic foot patients, there is higher prevalence of cardiovascular events [29]. As an additional cardiovascular risk factor, several hypothetical pathogenic etiologies of diabetic foot, including inflammatory events which may be related to elevated plasma interleukin-6 level and decline plasma adiponectin level are proposed [29, 30].

Increasing evidence has demonstrated the benefit of DPP4i therapy in protecting endothelial function [13, 31], including even the possibility of regressing carotid atherosclerosis [32]. In clinical practice, however, considerable debate exists [33]. Insulin has a powerful ability to promote glycaemic control and β-cell function preservation [34]. However, there seems to be increasing evidence that hyperinsulinemia might be associated with atherosclerosis and an increased risk of cardiovascular disease [8, 9, 35, 36].

We found a lower incidence of MACEs including nonfatal MI, nonfatal stroke, cardiac death, any death, amputation and heart failure for admission rate among the patients who received DPP4i-based therapy than in those treated with combined DPP4i- and insulin-based therapy. In addition, compared with the combined therapy users, the insulin-based users also had a lower incidence of these outcomes except for nonfatal stroke. In patients with poorly controlled diabetic foot, physicians often add other antidiabetic medicines with different mechanisms for better glycaemic control, however the effect is often limited in this population. The worse glucose control and comorbidities in these patients with diabetic foot may explain the higher incidence of cardiovascular disease in the combined therapy users. In addition, we observed a relatively lower HR for MACEs and diabetic cardiovascular complications in the DPP4i-based group than in the insulin-based group. Jil et al. [37] reported that adding insulin to metformin and sulfonylurea therapy was associated with an increased risk of cardiovascular events and death compared with adding DPP-4 inhibitors. This further supports the concept that DPP4i-based therapy has an endothelial protective effect which may reduce cardiovascular events. Even after propensity score matching in the three groups, we still observed a significantly lower incidence of MACEs, nonfatal MI, nonfatal stroke, cardiac death, death resulting from any cause, amputation and heart failure for admission rate in the DPP4i-based group than in the combined therapy group. Whereas, in the insulin-based group, only the incidence of death resulting from any cause and amputation decreased.

In our subgroup analysis, the endothelium-protective effect of DPP4i seemed to decline in the diabetic foot patients with ESRD. Atherosclerosis, vascular calcification, and stiffening are major risk factors for cardiovascular disease in patients with ESRD, and they have been associated with multiple mechanisms including endothelial dysfunction, inflammatory processes, oxidative stress, and calcium deposition in vascular tissue [38, 39]. Moreover, the process of arterial stricture and functional changes have been reported to be accelerated in patients with ESRD compared with normal control subjects [40]. This is compatible with our finding that in the diabetic foot patients with ESRD, the benefit of DPP4i in reducing the rate of cardiovascular disease disappeared.

In further comparisons of MACEs between the DPP4i-based and combined therapy groups (basal insulin or non-basal insulin), DPP4i-based treatment was most beneficial for MACE outcomes, where as combined therapy with basal insulin was not beneficial for MACE outcomes. The worst MACE outcome was found in those treated with insulin-only therapy with non-basal insulin. For advanced glycaemic control, physicians tend to prescribe insulin therapy with a basal-bolus regimen or premixed insulin analogues for diabetic patients with high glycemic variability. However, in patients with diabetic foot complications, adequate glucose control is harder to achieve than in other diabetic patients without such comorbidities [41]. Moreover, the risk of hypoglycemia and associated variations in glucose have been reported to be higher with the use of non-basal insulin [42], especially in patients with infection who have unstable diabetic foot complications. The higher the glycemic variability in patients with diabetes mellitus, the higher the oxidative stress produced [43]. In addition, postprandial hyperglycemia has been proposed to be highly associated with the macrovascular complications of diabetes [44]. In contrast, the combination of DPP4i with basal insulin has been reported to typically produce a more robust postprandial glucose-lowering effect and lower risk of hypoglycemia than the combination with other types of insulin [45]. Furthermore, several studies have reported that DPP4i therapy can benefit diabetic foot ulcer healing [46–48]. We suggest that for patients with diabetic foot, there is no need for additional insulin therapy for those who are already receiving DPP4i-based therapy in terms of cardiovascular outcomes.

This study has several limitations. First, because alogliptin was introduced after 2014 in Taiwan, not all DPP4i were studied. Second, this was a nonrandomized, retrospective, observational study, and thus selection bias is possible despite comprehensive propensity score matching and setting the index date as the start of therapy. However, diabetic foot patients are a thigh risk of cardiovascular disease, and these confounding factors could be minimized by our large cohort size and adequate follow-up period of at least 1 year. Third, dosages of the studied medications were not considered in the model because it would have further complicated the already complex model and led to a low matching rate between groups. Fourth, subjects who were suggested to take both insulin and DPP4i therapy were probably those more complicated from a clinical point of view. In this retrospective study, we could not definitely rule out such a circumstance, however we could still analyze the cardiovascular outcomes influenced by the effects of antidiabetic drugs beyond glycaemic control. Fifth, data of glycaemic control was lacking in this database although it is a strong confounding factor in cardiovascular outcomes. Sixth, some details of diabetic foot including the PEDIS classification for the wound could not be distinguished by ICD-9 codes. The DM foot status could have impact on the patients’ outcomes. However, for the majority of diabetic foot patients, we could still assess the effects of DPP4i- and add-on insulin-based therapy on cardiovascular outcomes in such a large cohort study.

## Conclusions

In conclusion, we found that in patients treated with DPP4i-based therapy and insulin-based, there were an association with lower risk of cardiovascular complications compared to patients treated with coadministration of insulin and DPP4i therapy, but this effect disappeared in those with concurrent ESRD. With regards to the clinical implication, there was no additional benefit in reducing the risk of cardiovascular events by adding insulin to DPP4i-based therapy for the patients with diabetic foot.

## Additional files

**Additional file 1: Table S1.** ICD-9-CM code details.

**Additional file 2: Table S2.** Characteristics at baseline for diabetic foot ulcer population, original data.

**Additional file 3: Table S3.** Characteristics at baseline of the patients with diabetic foot ulcers, by propensity score matching.

## Abbreviations

CI: confidence interval
DPP-4: dipeptidyl peptidase-4
ESRD: end-stage renal disease
GLP-1: glucagon-like peptide-1
HR: hazard ratio
MACE: major adverse cardiovascular event
MI: myocardial infarction
NHIRD: National Health Insurance Research Database
HbA1c: glycated hemoglobulin
