# Supplementary material for: Coadministration of DPP-4 inhibitor and insulin therapy does not further reduce the risk of cardiovascular events compared with DPP-4 inhibitor therapy in diabetic foot patients: a nationwide population-based study
Source: Diabetol Metab Syndr. 2018 Oct 17;10:75. doi: 10.1186/s13098-018-0378-6 (PMC6192159; doi:10.1186/s13098-018-0378-6)
Supplement: Supplementary file 1 — Additional file 1: Table S1. ICD-9-CM code details. [file 13098_2018_378_MOESM1_ESM.docx]

Table S1 ICD-9-CM code details

| Code | Code information |
| --- | --- |
| 250.70 | diabetes with peripheral circulatory disorders |
| 040.0 | gas gangrene |
| 785.4 | gangrene |
| 440.2 | atherosclerosis of native arteries of the extremities |
| 440.21 | atherosclerosis of native arteries of the extremities with intermittent claudication |
| 440.22 | atherosclerosis of native arteries of the extremities with rest pain |
| 440.23 | atherosclerosis of native arteries of the extremities with ulceration |
| 730.07 | acute osteomyelitis, ankle and foot |
| 730.17 | chronic osteomyelitis, ankle and foot |
| 730.27 | unspecified osteomyelitis, ankle and foot |
| 730.97 | unspecified infection of bone, ankle and foot |
| 707.14 | ulcer of the heel and midfoot |
| 707.15 | ulcer of other parts of the foot |
| 707.1 | ulcer of lower limbs, except pressure ulcer |
| 680.7 | carbuncle and furuncle of the foot |
| 682.7 | cellulitis and abscess of the foot, except toes |
| 681.1 | cellulitis and abscess of toes |
| 681.10 | cellulitis and abscess of toes, unspecified |
| V49.71 | Great toe amputation status |
| V49.72 | other toe(s) amputation status |
| V49.73 | foot amputation status |
| V49.74 | ankle amputation status |
| V49.75 | below the knee amputation status |
| V49.76 | Above the knee amputation status |
| V49.77 | hip amputation status |
| V52.1 | fitting and adjustment of artificial leg (complete or partial) |
| 84.11 | amputation of a toe |
| 84.12 | amputation through the foot |
| 84.13 | disarticulation of the ankle |
| 84.14 | amputation of the ankle through malleoli of the tibia and fibula |
| 84.15 | other amputation below the knee |
| 84.16 | disarticulation of the knee |
| 84.17 | amputation above the knee |

Abbreviation: ICD-9-CM: International Classification of Diseases, 9^th^ Revision, Clinical Modification
