# Supplementary material for: Coadministration of DPP-4 inhibitor and insulin therapy does not further reduce the risk of cardiovascular events compared with DPP-4 inhibitor therapy in diabetic foot patients: a nationwide population-based study
Source: Diabetol Metab Syndr. 2018 Oct 17;10:75. doi: 10.1186/s13098-018-0378-6 (PMC6192159; doi:10.1186/s13098-018-0378-6)
Supplement: Supplementary file 2 — Additional file 2: Table S2. Characteristics at baseline for diabetic foot ulcer population, original data. [file 13098_2018_378_MOESM2_ESM.docx]

| Table S2 Characteristics at Baseline for Diabetic foot ulcer population, original data | | | | | |
| --- | --- | --- | --- | --- | --- |
| Demographic characteristic | Total | Combine use | DPP-4 inhibitors | Insulin only | P value |
| Population, n (%) | 19791 | 11400 | 6466 | 1925 | - |
| Men, n (%) | 10136 (51.2 ) | 5887 (51.6 ) | 3255 (50.3 ) | 994 (51.6 ) | 0.2296 |
| Age, y, mean ±SD | 58.77±12.47 | 58.8±12.8 | 58.8±11.7 | 58.5±13.0 | 0.5182 |
| Age group, y, n (%) |  |  |  |  | <.0001 |
| 20-29 | 211 (1.1) | 132 (1.2) | 49 (0.8) | 30 (1.6) |  |
| 30-39 | 1041 (5.3) | 663 (5.8) | 266 (4.1) | 112 (5.8) |  |
| 40-49 | 3255 (16.4) | 1878 (16.5) | 1044 (16.1) | 333 (17.3) |  |
| 50-59 | 6142 (31) | 3420 (30) | 2143 (33.1) | 579 (30.1) |  |
| 60-69 | 4982 (25.2) | 2801 (24.6) | 1738 (26.9) | 443 (23.0) |  |
| 70-79 | 3265 (16.5) | 1934 (17) | 1000 (15.5) | 331 (17.2) |  |
| >=80 | 895 (4.5) | 572 (5) | 226 (3.5) | 97 (5.0) |  |
| Comorbidity |  |  |  |  |  |
| Hypertension | 12200 (61.6) | 7048 (61.8) | 4066 (62.9) | 1086 (56.4 ) | <.0001 |
| Hyperlipidemia | 9048 (45.7) | 5065 (44.4) | 3265 (50.5) | 718 (37.3 ) | <.0001 |
| Nephropathy | 3550 (17.9) | 2456 (21.5) | 827 (12.8) | 267 (13.9 ) | <.0001 |
| Retinopathy | 1174 (5.9) | 853 (7.5) | 208 (3.2) | 113 (5.9 ) | <.0001 |
| Peripheral neuropathy | 2839 (14.3) | 1910 (16.8) | 686 (10.6) | 243 (12.6 ) | <.0001 |
| ESRD | 978 (4.9) | 806 (7.1) | 114 (1.8) | 58 (3.0 ) | <.0001 |
| Peripheral artery disease | 5758 (29.1) | 3385 (29.7) | 1878 (29) | 495 (25.7 ) | 0.0018 |
| Medication history |  |  |  |  |  |
| Antithrombotic drug | 6437 (32.5) | 4137 (36.3) | 1666 (25.8) | 634 (32.9 ) | <.0001 |

Abbreviation: ESRD: end-stage renal disease
