# Supplementary material for: Coadministration of DPP-4 inhibitor and insulin therapy does not further reduce the risk of cardiovascular events compared with DPP-4 inhibitor therapy in diabetic foot patients: a nationwide population-based study
Source: Diabetol Metab Syndr. 2018 Oct 17;10:75. doi: 10.1186/s13098-018-0378-6 (PMC6192159; doi:10.1186/s13098-018-0378-6)
Supplement: Supplementary file 3 — Additional file 3: Table S3. Characteristics at baseline of the patients with diabetic foot ulcers, by propensity score matching. [file 13098_2018_378_MOESM3_ESM.docx]

| Table S3 Characteristics at baseline of the patients with diabetic foot ulcers, by propensity score matching | | | | | | |
| --- | --- | --- | --- | --- | --- | --- |
| Demographic data | Combine use | DPP-4i | P value | Combined use | Insulin only | P value |
| Population, n (%) | 6185 | 6185 | - | 1874 | 1874 | - |
| Men, n (%) | 3123 (50.5) | 3125 (50.5) | 0.9713 | 952 (50.8) | 967 (51.6) | 0.6240 |
| Age, y, mean ±SD | 59.1±12.8 | 58.8±11.7 | 0.1393 | 58.7±12.9 | 58.6±13.5 | 0.9127 |
| Age group, y, n (%) |  |  | <0.0001 |  |  | 0.8847 |
| 20-29 | 66 (1.1) | 48 (0.8) |  | 25 (1.3) | 30 (1.6) |  |
| 30-39 | 350 (5.7) | 258 (4.2) |  | 99 (5.3) | 106 (5.7) |  |
| 40-49 | 986 (15.9) | 1007 (16.3) |  | 330 (17.6) | 320 (17.1) |  |
| 50-59 | 1880 (30.4) | 2035 (32.9) |  | 563 (30) | 562 (30) |  |
| 60-69 | 1493 (24.1) | 1667 (27) |  | 452 (24.1) | 430 (22.9) |  |
| 70-79 | 1077 (17.4) | 953 (15.4) |  | 305 (16.3) | 329 (17.6) |  |
| >=80 | 333 (5.4) | 217 (3.5) |  | 100 (5.3) | 97 (5.2) |  |
| Comorbidity |  |  |  |  |  |  |
| Hypertension | 3927 (63.5) | 3873 (62.6) | 0.3144 | 1070 (57.1) | 1073 (57.3) | 0.9211 |
| Hyperlipidemia | 3052 (49.3) | 3091 (50) | 0.4831 | 739 (39.4) | 713 (38) | 0.3833 |
| Nephropathy | 827 (13.4) | 825 (13.3) | 0.9578 | 251 (13.4) | 267 (14.2) | 0.4489 |
| Retinopathy | 211 (3.4) | 208 (3.4) | 0.8815 | 110 (5.9) | 113 (6) | 0.8359 |
| Peripheral neuropathy | 697 (11.3) | 680 (11) | 0.627 | 226 (12.1) | 243 (13) | 0.4013 |
| ESRD | 375 (6.1) | 110 (1.8) | <0.0001 | 137 (7.3) | 58 (3.1) | <0.0001 |
| Peripheral artery disease | 1813 (29.3) | 1802 (29.1) | 0.8278 | 536 (28.6) | 495 (26.4) | 0.1337 |
| Medication history |  |  |  |  |  |  |
| Antithrombotic drug | 1645 (26.6) | 1646 (26.6) | 0.9838 | 611 (32.6) | 627 (33.5) | 0.5784 |

Abbreviation: ESRD: end-stage renal disease
